# Antibacterial Potential of Gold Nanoparticles Synthesized From Leaf Extract of Syzygium cumini Against Multidrug-Resistant Urinary Tract Pathogens

**DOI:** 10.7759/cureus.34830

**Published:** 2023-02-10

**Authors:** Diksha Diksha, Shailesh K Gupta, Pratima Gupta, Uttam C Banerjee, Deepjyoti Kalita

**Affiliations:** 1 Microbiology, All India Institute of Medical Sciences, Rishikesh, IND; 2 Biotechnology, Amity University, Mohali, IND

**Keywords:** minimum inhibitory concentration, urinary tract pathogens, leaf extract, syzygium cumini, multidrug-resistant bacteria, medicinal plants, gold nanoparticle, urinary tract infection

## Abstract

Background: Urinary tract infection (UTI) is one of the most commonly encountered bacterial infections. Due to the misuse or excessive use of antibiotics, the upsurge of multidrug-resistance cases in UTIs has now become a global threat to public health. Exploring a newer or safer treatment using green synthesized nanoparticles (NPs) is another substitute for eliminating multidrug-resistant pathogens.

Methodology: Leaf extract of *Syzygium cumini* was used for green synthesis of gold NPs. Synthesis of *Syzygium cumini *gold nanoparticles (ScAu-NPs) was achieved by optimizing various reaction parameters. These ScAu-NPs were characterized through UV-visible spectroscopy, transmission electron microscope, Fourier transform infrared spectroscopy (FTIR), and X-ray diffraction. ScAu-NPs were then processed for antibacterial activity against clinically isolated multidrug-resistant pathogens like *Escherichia coli*, *Klebsiella pneumoniae, Proteus vulgaris, Acinetobacter baumannii, Staphylococcus aureus*, and *Enterococcus faecalis*.

Results: Characterization of NPs revealed that biosynthesized NPs were spherical in shape. FTIR spectroscopy showed the presence of phenolics and aromatic compounds. Biosynthesized NPs exhibit good antibacterial activity with a significant bacterial reduction seen against all bacterial isolates compared to the controls.

Conclusion: From the results of the present study, the formulation of biosynthesized ScAu-NPs can be utilized in drug development for eliminating infections caused by multidrug-resistant pathogens.

## Introduction

Urinary tract infections (UTIs) are one of the most common bacterial infections that contribute significantly to mortality and morbidity by affecting 150 million individuals annually [1]. Misuse or exploitation of antibiotics in the treatment of UTIs has led to drug resistance in pathogens globally [2]. A higher level of resistance can be seen in *Enterobacteriaceae* members of uropathogens, which produce enzymes like carbapenemase and extended-spectrum β-lactamases (ESBL) [3]. A large number of multidrug resistance cases of UTI were also reported in India [4]. To tackle the issue of resistance, researchers have drawn their attention to nanotechnology. Nanoparticle (NP)-based therapy is now been considered an alternative treatment for UTIs [5]. Silver, copper, titanium, zinc, gold, and magnesium are the most commonly used elements involved in NP synthesis; among all, gold nanoparticles (AuNPs) are used widely in biomedical applications due to their good optical properties [6]. According to previous studies, small-sized AuNPs significantly assist in antibacterial activity [7,8]. Plant-mediated route of NP synthesis is less toxic for humans and is environmentally friendly [9,10]. Therefore, in this study, we synthesized AuNPs using the leaf extracts of the plant *Syzygium cumini*. Essential oil of the leaves of *S. cumini* already showed substantial antioxidant and antibacterial activity [11]. Therefore, the leaf extract of this plant has been used as a reducing agent for AuNP synthesis by optimizing its reaction parameters. The present study also aimed to explore the antibacterial activity of biosynthesized *S. cumini* gold nanoparticles (ScAu-NPs) and also investigate the minimum inhibitory concentration (MIC) of ScAu-NPs required to inhibit the growth of multidrug-resistant uropathogens.

## Materials and methods

Experimental setup

Synthesis of Gold Nanoparticles

Leaves of *S. cumini* were washed, shed dried at room temperature, and ground to obtain a fine powder. Ten grams of leaves were dissolved in distilled water, sonicated, and filtered followed by lyophilization. Synthesis of AuNPs was achieved by optimizing the reaction parameters such as concentration of salt and plant extract, temperature, reaction time, and pH. After optimizing, these NPs were then characterized through UV-visible spectroscopy (UV-Vis) (Agilent Technologies, Santa Clara, California). The shape and size of NPs were estimated by transmission electron microscopy (TEM) (FEI Company, Hillsboro, Oregon), and crystalline nature was detected through X-ray diffraction (XRD) patterns and selected area electron diffraction (SAED) (Rigaku Miniflex 600 X-ray Diffractometer, Rigaku Corporation, Tokyo, Japan) with a 2θ range of 5-90°. The functional group was detected through Fourier transform infrared spectroscopy (FTIR) (PerkinElmer, Waltham, Massachusetts) with 400-4000 cm^-1^.

Antimicrobial Susceptibility of Bacterial Isolates and Antibacterial Activity of AuNPs

Bacterial isolates were isolated from 200 mid-stream urine samples. These isolates were identified through biochemical tests, and antimicrobial susceptibility testing was performed. Antibacterial activity was accomplished through the Kirby-Bauer disk diffusion method. To test the antibacterial activity of ScAu-NPs, the bacterial inoculum was grown in Luria-Bertani (LB) broth and adjusted to 0.5 McFarland. ScAu-NPs were impregnated into the sterile disc in Mueller Hinton agar (MHA) plates. Quality control of the MHA medium was performed before performing the experiments. These plates were then incubated at 37°C, followed by a measurement of the zone of inhibition. MIC was calculated using the micro-broth dilution method (CLSI M07-A8). All bacterial inoculums were treated with different concentrations (100, 150, 250, 350, 450, and 500 µg/mL) of ScAu-NPs and incubated at 37°C. MIC was then calculated through visual observation and bacterial absorbance at an optical density (OD) of 600 nm.

## Results

Characterization of gold nanoparticles

**Figure 1:**
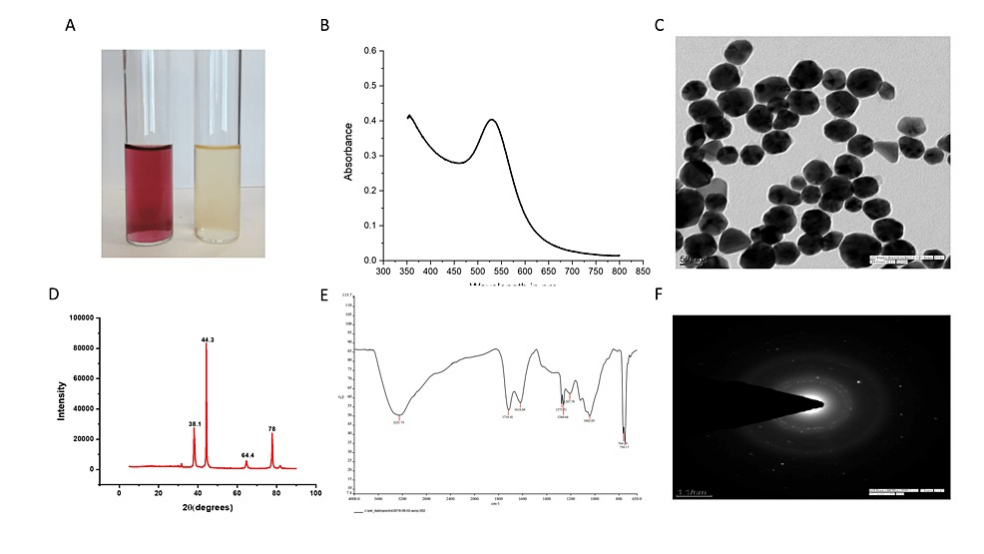
Synthesis and characterization of AuNPs using the leaf extract of Syzygium cumini. (A) Preliminary identification of AuNP synthesis by changing the color of the solution from yellow to purple-red. (B) The UV-visible spectrum of the optimized parameter of biosynthesized AuNPs forming a bell shape curve under the wavelength of 520-535 nm further confirms its synthesis. (C) Characterization of AuNPs through transmission electron microscopy that showed spherical-shaped nanoparticles with an average size of 50-60 nm in diameter. (D) FTIR spectrum of biosynthesized AuNPs that shows the possible functional group that acts as a reducing agent for AuNP synthesis. (E) XRD pattern of AuNPs that displays the crystallinity of nanoparticles and the diffraction peaks that corresponds to the planes of gold atoms. (F) SAED pattern of AuNPs further confirms the crystallinity by exhibiting the electron diffraction pattern of AuNPs. AuNPs: gold nanoparticles; FTIR: Fourier transform infrared spectroscopy; XRD: X-ray diffraction; SAED: selected area electron diffraction.

Preliminary identification of the biosynthesis of AuNPs was made through color change. A quick change in color from yellow to purple-red indicates the synthesis of AuNPs and it gets intensified over time due to surface plasmon resonance (SPR) phenomena (Figure 1A).

Reaction parameters like plant extract, salt concentration, pH, time, and temperature were optimized for ScAu-NPs synthesis. Results of the optimization of reaction parameters showed that a higher yield of NPs was observed at a 1 mM concentration of salt with 200-500 ul of plant extract in an acidic pH at a temperature of 30-600°C. This optimized solution was subjected to UV-Vis spectroscopy that showed the formation of a bell-shaped curve at 520-535 nm wavelength (Figure 1B).

TEM was carried out to determine the morphology and size of the biosynthesized ScAu-NPs. Briefly, a drop of AuNP solution is placed in a copper grid and the grid is allowed to dry completely. It was noticed that biosynthesized ScAu-NPs were predominantly spherical in shape with an approximate size of 50-60 nm in size measured through ImageJ software (National Institutes of Health, Bethesda, Maryland) (Figure 1C).

FTIR analysis was performed to identify the possible functional group that assists in NP synthesis. Organic extracts interact with metal salts and thereby facilitate NP synthesis [12]. In our study, *S. cumini* leaf extracts were used as a reducing agent for AuNP synthesis. FTIR spectrum showed absorption bands at 3251.75, 1718.41, 1618.84, 1275.51, 1260.64, 1207.79, 1042.85, 764.29, and 750.17 cm^-1^, which corresponds to 0-H, C-H, C=O, and C=C stretching bands that indicate the presence of carboxylic acid, aromatic compounds, phenolic, conjugated alkene, and aryl ether functional groups in the biosynthesized AuNPs (Figure 1D).

XRD provides information about the crystallinity of the NPs. Our results clearly depict the crystalline nature of AuNPs by forming sharp and clear peaks with faces at different 2θ values as shown in Figure 1E. Biosynthesized AuNPs show four diffraction peaks with 2θ values at 38.10, 44.30, 64.40, and 77.50, corresponding to the crystal planes 111, 220, and 311. These planes are indexed with metallic gold. The crystal planes also confirm the face-centered cubic (FCC) structure. The crystallinity of AuNPs was also confirmed through the SAED pattern that shows diffraction rings indexed with the above planes of AuNPs (Figure 1F).

Antibiotic susceptibility testing

Results of antibiotic susceptibility testing of isolated strains causing UTI showed resistance to various classes of antibiotic drugs such as cephalosporin, carbapenem, nitrofurantoin, aminoglycosides, lincosamides, tetracyclines, sulfonamide, quinolone, and monobactam (Table 1).

**Table 1:** Antibiotic susceptibility pattern of multidrug-resistant pathogens causing urinary tract infection. CTX: cefotaxime; CXM: cefuroxime; CTR: ceftriaxone; CPM: cefepime; CAZ: ceftazidime; CX: cefoxitin; DOR: doripenem; MRP: meropenem; ETP: ertapenem; IMP: imipenem; NIT: nitrofurantoin; AK: amikacin; GEN: gentamicin; TOB: tobramycin; CIP: ciprofloxacin; CD: clindamycin; DOX: doxycycline; COT: cotrimoxazole; NX: norfloxacin; AZ: aztreonam; AZM: azithromycin; E: erythromycin; LZ: linezolid; VA: vancomycin; TE: tetracycline; PTZ: piperacillin-tazobactam.

		Susceptibility pattern																																				
S. No.	Microorganisms	Cephalosporins						Carbapenem				Nitrofuran	Aminoglycosides			Fluoroquinolones		Lincosamides		Tetracyclines		Sulfonamide		Quinolone		Monobactam		Macrolides			Oxazolidinones		Glycopeptide		Tetracycline		Penicillins	
		CTX	CXM	CTR	CPM	CAZ	CX	DOR	MRP	ETP	IMP	NIT	AK	GEN	TOB		CIP		CD		DOX		COT		NX		AZ		AZM	E		LZ		VA		TE		PTZ
1	Escherichia coli	R	R	R	R	-	-	S	S		R	S	R	R	R		R		-		-		R		R		-		-	-		-		-		-		R
2	Klebsiella pneumoniae	R	R	R	R	-	-	R	R	R	R	R	R	R	R		R		-		-		R		R		-		-	-		-		-		-		R
3	Proteus vulgaris	R	R	R	R	-	-	S	S	R	R	S	S	R	R		R		-		-		S		R		-		-	-		-		-		-		R
4	Acinetobacter baumannii	R	-	R	R	R	-	R	R	-	-	R	R	R	R		-		-		-		-		R		-		-	-		-		-		-		-
5	Pseudomonas aeruginosa	-	-	-	R	R	-	S	R	-	R	R	R	R	R		R		-		-		R		R		-		-	-		-		-		-		R
6	Staphylococcus aureus	-	-	-	-	-	R	-	-	-	-	R	-	-	-		-		R		-		-		R		-		R	R		S		-		R		-
7	Enterococcus faecalis	-	-	-	-	-	-	-	-	-	-	R	-	R	-		-		-		-		-		R		-		-	-		S		R		R		-

Antibacterial activity and MIC of ScAu-NPs

**Figure 2:**
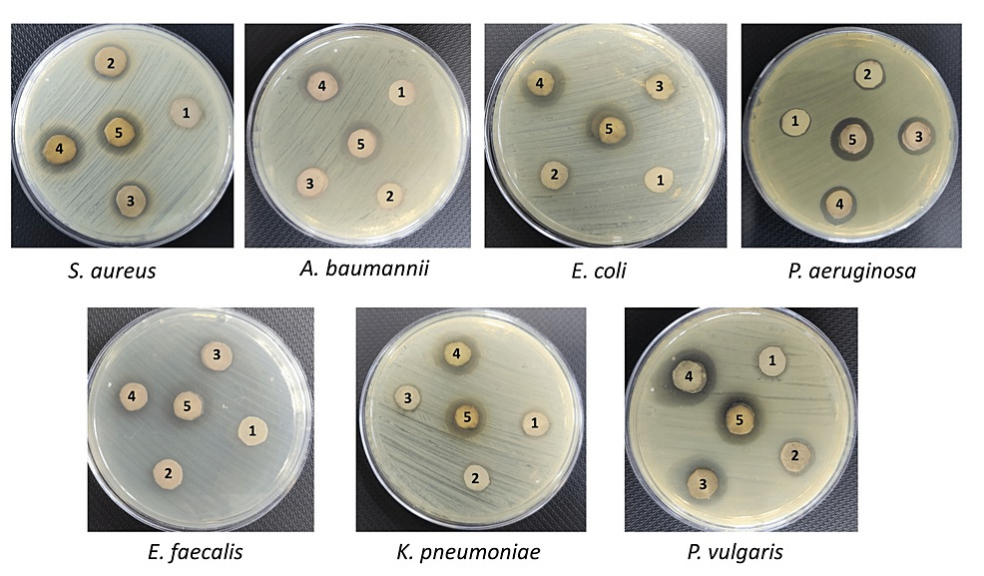
Antibacterial activity of AuNPs through disk diffusion. Antibacterial activity of AuNPs by forming zone of inhibition against Staphylococcus aureus, Acinetobacter baumannii, Escherichia coli, Pseudomonas aeruginosa, Enterococcus faecalis, Klebsiella pneumonia, and Proteus vulgaris at various concentrations. Zone diameter increases by increasing the concentrations (25, 50, 100, 150, and 200 µg/mL) of AuNPs. 1 - 25 µg/mL, 2 - 50 µg/mL, 3 - 100 µg/mL, 4 - 150 µg/mL, and 5 - 200 µg/mL. AuNPs: gold nanoparticles.

In this study, the antibacterial activity of biosynthesized ScAu-NPs was checked through the disk diffusion method against seven multidrug-resistant microorganisms, i.e., *Staphylococcus aureus*, *Acinetobacter baumannii*, *Escherichia coli*, *Pseudomonas aeruginosa*, *Enterococcus faecalis*, *Klebsiella pneumoniae*, and *Proteus vulgaris*. Results of disk diffusion displayed the significant antibacterial activity of ScAu-NPs by inhibiting the growth of all pathogens. An increase in zone diameter was observed when increasing ScAu-NPs concentrations (25, 50, 100, 150, and 200 µg/mL). The average zones of inhibition formed by ScAu-NPs against *S. aureus*, *A. baumannii*, *E. coli*, *P. aeruginosa*, *E. faecalis*, *K. pneumoniae*, and *P. vulgaris* are 17.87 mm, 17.69 mm, 17.65 mm, 17.79 mm, 17.65 mm, 16.7 mm, and 20.05 mm, respectively, which display considerable antibacterial activity of AuNPs, as shown in Figure 2.

**Figure 3:**
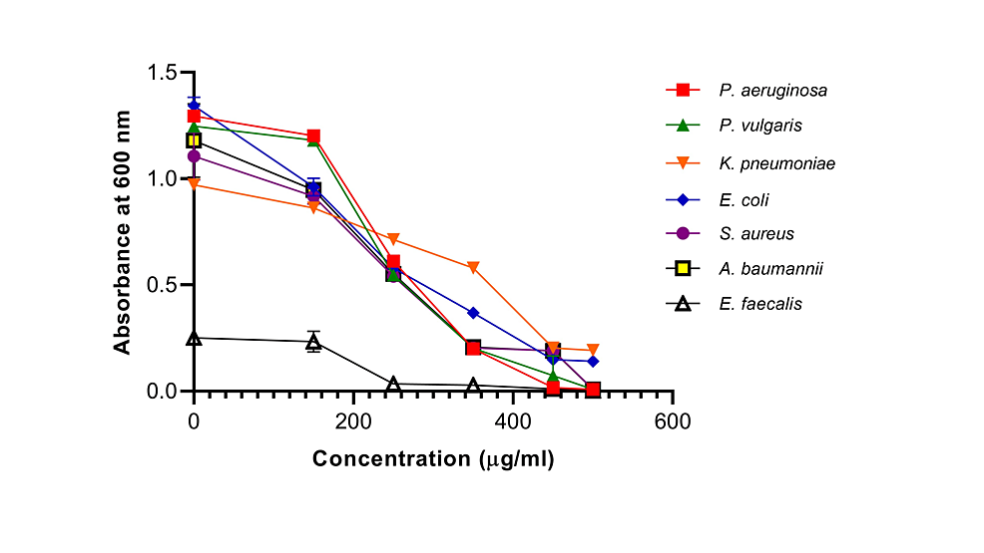
Antibacterial activity of AuNPs determined through minimum inhibitory concentration (MIC). Various concentrations of AuNPs like 100, 150, 250, 350, 450, and 500 µg/mL were used. A decline in the graph of each pathogen (designated through symbols) was observed at increasing concentrations of AuNPs and thereby calculated MIC through a reduction in OD value at 600 nm. AuNPs: gold nanoparticles; OD: optical density.

From the above findings of the disk diffusion assay, it is evident that ScAu-NPs exhibit considerable antibacterial activity. To see at which concentration ScAu-NPs inhibit bacterial growth, MIC was also checked against these pathogens. MIC against all uropathogens was determined through visual observation and absorbance at OD 600 nm. It was observed that no growth was seen at a concentration of 350 µg/mL for *P. vulgaris*, *P. aeruginosa*, *S. aureus*, and *A. baumannii*. MICs of *E. coli* and *K. pneumoniae* were achieved at 450 µg/mL and 250 µg/mL for *E. faecalis* with a sharp reduction in OD when compared to positive control. MIC of ScAu-NPs against all multidrug-resistant uropathogens can be seen in Figure 3. A decline in bacterial growth of isolated strains was observed when the concentration of ScAu-NPs increases.

## Discussion

UTI has now become a challenge in a hospital setting due to the exploitation or overuse of antibiotics in the treatment, which led to the emergence of multidrug resistance in urinary tract pathogens [2]. To overcome this load of multidrug resistance, scientists and researchers are constantly seeking other therapeutic agents as an alternative to traditional antibiotics. AuNPs exhibit enormous biomedical application that includes antibacterial, antifungal, and anti-cancer activities, and due to their intrinsic properties, they are now widely used in drug delivery and diagnosis [6]. Plant extracts served as an excellent reducing agent for AuNP synthesis due to the presence of active phytochemicals and they also favor a greener and safer route of NP synthesis in causing fewer side effects compared to chemically synthesized NPs [13,14]. *S. cumini*, also known as Jamun, is a tropical evergreen tree in India and previous reports have shown the antibacterial activity of the leaf extract of this plant against multidrug-resistant strains [15]. Therefore, leaves of *S. cumini* were used to synthesize AuNPs. From our results, it is evident that the leaf extract of *S. cumini* reduces the gold ions by changing the color of the solution into a reddish-purple that indicates the synthesis of AuNPs [16]. The formation of ScAu-NPs was further confirmed through UV-Vis spectroscopy by forming an SPR band at around 530 nm. SPR peak in the range of 527-535 nm is attributed to spherical-shaped NPs with a size of approximately 30-50 nm and concordant with our TEM images. Images of TEM showed synthesized NPs were spherical in shape, and well distributed with no evidence of aggregation [17,18]. The presence of hydroxyl, flavonoids, carboxyl, and phenolics groups in the FTIR spectrum showed the possible functional group that helped in stabilizing and capping of NPs, as reported in a previous study [19].

In context with the current scenario of multidrug resistance in uropathogens, researchers have synthesized NPs using the plant as a reducing agent. In this study, leaves of *S. cumini* were used as a reducing agent for the synthesis of AuNPs to see their efficacy against multidrug-resistant urinary tract pathogens. A similar study synthesized silver NPs using the inflorescence of *Tridax procumbens* and concluded their medicinal properties in inhibiting multidrug-resistant urinary tract isolates [20]. Few studies have also synthesized silver, gold, and iron NPs using the leaf extract of *S. cumini* but their NP formulation against multidrug-resistant urinary tract pathogens is yet to be explored [21,22]. The antimicrobial effect of zinc NPs was also reported in a study, which concluded that zinc NPs inhibit the biofilm formation of urinary tract-infected pathogens [23]. Our findings of antibacterial activity revealed that escalating the concentrations of ScAu-NPs increases the zone diameter. Similar findings were observed by other researchers when they examined the antibacterial activity of plant-mediated AuNPs against clinical and American Type Culture Collection (ATCC) strains and concluded substantial antibacterial activity [24,25]. MIC of ScAu-NPs was also calculated to determine the least concentration that inhibits the growth of uropathogens. Our results showed that all pathogens displayed growth at <250 µg/mL AuNP concentration but further increasing the concentration brings down the bacterial growth when measured at OD 600 nm. Hence, a greener mode of AuNP synthesis can be employed or explored in drug development formulation, as the greener route of synthesis provides quick, eco-friendly, and effective ways to eliminate UTIs caused by multidrug-resistant pathogens [26].

There are a few limitations of the study. Though this study showed satisfactory and good antibacterial activity of AuNPs against multidrug resistance but it is also constrained in assessing the possible phytochemicals present in the leaf extract of *S. cumini* that aid in AuNP synthesis. Also, this is a complete in vitro study and provides a foundation for newer drug development by bringing out antimicrobial compounds from *S. cumini* to treat bacterial infections caused by these multidrug-resistant strains. Still, in vivo studies are further required to see its potency on animal models.

## Conclusions

The existing situation of the emergence of multidrug resistance in urinary tract pathogens led researchers to seek an alternative therapeutic agent. Nanotechnology is itself an asset in the biomedical field and is widely used in drug delivery, drug development, and diagnostics. Therefore, AuNPs were used as an antibacterial agent against multidrug-resistant urinary tract pathogens. In this study, the greener approach of ScAu-NPs synthesis showed significant antibacterial activity against multidrug-resistant pathogens. These biosynthesized ScAu-NPs inhibit the growth of all multidrug-resisting uropathogens. To the best of the authors' knowledge, this is the first report that checked the antibacterial activity of AuNPs against multidrug resistance of UTI using the leaf extract of *S. cumini*. Hence, further drug development by incorporating biosynthesized AuNPs could be a useful tool for eliminating the UTI infection caused by multidrug resistance.
